# Beyond hypertrophy: a network physiology perspective on the cardio-neuromuscular trade-off in elite soccer

**DOI:** 10.3389/fnetp.2026.1741770

**Published:** 2026-02-13

**Authors:** Zacharias Papadakis, Nikolaos Koutlianos, Vassilios Panoutsakopoulos, Evangelia Kouidi

**Affiliations:** 1 Department of Health Sciences and Clinical Practice, College of Health Professions and Medical Sciences, Barry University, Miami Shores, FL, United States; 2 Laboratory of Sports Medicine, School of Physical Education and Sport Science at Thessaloniki, Aristotle University of Thessaloniki, Thessaloniki, Greece; 3 Biomechanics Laboratory, School of Physical Education and Sport Science at Thessaloniki, Aristotle University of Thessaloniki, Thessaloniki, Greece

**Keywords:** athlete’s heart, concurrent training, left ventricular mass index, network method and edges, sensitivity findings, vertical jump, mechanical power, sports performance

## Abstract

**Introduction:**

Conventional models treat cardiovascular and neuromuscular adaptations as independent, which can hide interference between endurance and power. We investigated whether cardiac remodeling is associated with peak explosive power when adaptation is considered as an integrated system.

**Methods:**

Nineteen male Super League soccer players completed two-dimensional echocardiography to quantify left ventricular mass index (LVMI) and performed a fifteen-repetition vertical jump test. We adjusted variables for body size and training years, then estimated a partial-correlation network with a Gaussian graphical model and ran sensitivity and subgroup checks.

**Results:**

The developed network was sparse and stable. A selective inverse association linked LVMI with maximal jump height (partial correlation –0.41), supported by a complementary Bayesian analysis (Bayes factor 5.70). Neuromuscular variables formed a tight positive cluster, and LVMI did not show negative coupling with other jump metrics, indicating a specific rather than global trade-off.

**Discussion:**

In elite players, a cardiac phenotype consistent with endurance support coincided with constrained peak explosive output when the system was analyzed as a whole. An interdependent network view clarifies interference patterns and points to targeted monitoring and periodization strategies for high-performance sport.

## 1 Introduction

Elite professional soccer imposes a dualistic stimulus on players’ physiological system. Players must sustain prolonged aerobic efforts, which often involve covering more than 10 km per match, while also executing frequent, explosive anaerobic bursts such as sprints, jumps, and rapid changes of direction (Boraczynski et al., 2020; Nobari et al., 2023). As such, this unique hybrid tax on players’ physiology demand profound and simultaneous adaptations across both the cardiovascular and neuromuscular domains (Hostrup and Bangsbo, 2023; Morrison et al., 2023). Chronically structured training elicits measurable adaptations in both physiological domains (Francavilla et al., 2018; Green et al., 2024).

Regarding the cardiovascular related adaptations and the “Morganroth hypothesis,” the high-volume running produces a persistent volume overload that leads to eccentric left ventricular hypertrophy (LVH) and enhanced stroke volume, in contrast to the intermittent high-intensity activities that create a transient pressure overload that stimulates concentric hypertrophy (Churchill et al., 2021; Pelliccia et al., 2023; Pelliccia et al., 2025; Johnson et al., 2023; Pittaras et al., 2023; Starekova et al., 2020; von Lueder et al., 2018; Lewis et al., 2012). This adaptation culminates in the well-documented “athlete’s heart” (Baba Ali et al., 2024; Petek et al., 2022) manifested as an increase in left ventricular mass (LVM), which, when indexed to body surface area (LVMI), provides a metric that contextualizes the magnitude of cardiac remodeling, though distinguishing physiological from pathological hypertrophy requires a comprehensive assessment of cardiac function (Baba Ali et al., 2024; Oxborough et al., 2024; Unnithan et al., 2024; Albaeni et al., 2021). Evidence indicates that this adaptation is dose-dependent manifested as increases in LVMI in response to both recreational and elite-level soccer training (Unnithan et al., 2024; Unnithan et al., 2018; Sjuretharson et al., 2024). Contemporary practice scales mass to body size using the left ventricular mass index, with a commonly applied clinical threshold of about 115 g·m^−2^ to contextualize physiological hypertrophy (Oxborough et al., 2024). It is apparent that soccer training induces cardiac remodeling that can elevate LVMI over time. Although such remodeling can support stroke volume and oxygen delivery, elements important for an enhanced soccer performance, a larger myocardial mass may add inertial load that could hinder rapid neuromuscular actions required for explosive tasks (Pelliccia et al., 2025; Kandels et al., 2025; Deely et al., 2022; Di et al., 2024; Silvestrini et al., 2023).

Concurrently with the adaptations of the cardiovascular system, the neuromuscular system adapts to the demands of elite soccer play and training for explosive power. Soccer play and training involve repetitive vertical accelerations and decelerations which rely on the efficiency of the stretch-shortening cycle (SSC), a key component of anaerobic power performance (Suarez-Arrones et al., 2020; Akyildiz et al., 2022; Bishop et al., 2022; Stratford et al., 2020; Theodorou et al., 2013). These explosive capabilities relevant to soccer can be captured with a repetitive vertical jump test (RVJT) on a force plate (Theodorou et al., 2013). Metrics obtained from RVJT, such as maximum (h_MAX_) and average jump height (h_AVE_), reactive strength indices, and relative power exhibit strong reliability. Furthermore, these variables are associated with sprint and change-of-direction performance and can serve as effective discriminators of competitive standing among academy soccer players (Suarez-Arrones et al., 2020; Bishop et al., 2022; Stratford et al., 2020; Montalvo et al., 2021; Papadakis et al., 2022a; Bishop et al., 2023).

Historically, in the realm of sport and exercise science and cardiovascular exercise physiology the concepts of the concurrent training (CT) and the impact on the cardio-neuromuscular systems have been investigated in an isolated manner. In other words, the development of the “athlete’s heart” and the enhancement of explosive power with their adaptive pathways have been viewed as separate, parallel phenomena (Pelliccia et al., 2025; Di et al., 2025). Adopting this reductionist perspective overlooks a well-documented conflict in exercise physiology known as the “interference effect,” where simultaneously training for both endurance and strength can lead to compromised or attenuated adaptations, particularly in explosive power (Schumann et al., 2022; Vechin et al., 2021). Moreover, this siloed approach may represent a significant blind spot, as it overlooks the potential for functional trade-offs within the integrated physiological system (Pelliccia et al., 2025; Di et al., 2025; Berger et al., 2024; Furrer et al., 2023). During CT, when endurance and strength-power stimuli are combined, the training literature consistently identifies an interference pattern with the most pronounced attenuation in explosive tasks compared with maximal strength or endurance outcomes (Schumann et al., 2022; Huiberts et al., 2024; Manuel Clemente et al., 2021; Petre et al., 2021). Meta-analytic syntheses report that concurrent paradigms produce smaller gains in explosive strength than resistance-only programming and mixed results for maximal strength, reinforcing the plausibility of a functional trade-off affecting jump performance in soccer contexts (Akyildiz et al., 2022; Schumann et al., 2022; Manuel Clemente et al., 2021; Petre et al., 2021; Arslan et al., 2025). This potential antagonism, where profound endurance-type cardiac adaptations may functionally constrain peak anaerobic power, gives rise to what our team termed the cardio-neuromuscular performance paradox (Papadakis et al., 2025).

The Network Physiology of Exercise (NPE) framework possibly offers a more holistic lens to capture such a tradeoff, challenging the traditional approach by proposing that physiological systems do not operate in isolation but function as a complex, integrated network of dynamic interactions (Balagué et al., 2022; Balague et al., 2022; Balagué et al., 2024; Papadakis et al., 2022b; Papadakis et al., 2022c; Ivanov, 2021; Ivanov and Bartsch, 2014). From this perspective, the organism is a complex adaptive system where physiological states emerge from the specific organization and coupling of its various sub-networks (Balagué et al., 2022; Balague et al., 2022; Balagué et al., 2024; Balagué et al., 2020a; Ivanov et al., 2016). Therefore, the interference effect can be reframed under the NPE as systemic, network tradeoff, where the LVMI is not merely a measure of cardiac size but a node within a broader cardio-neuromuscular network. Its relationship with RVJT metrics, another key node, can reveal important information about the overall state and function of the athletic system.

No study has explicitly investigated this tradeoff as a functional coupling between a chronic, organ-level cardiovascular adaptation and a whole-body, task-specific neuromuscular output. The prevailing siloed approach has prevented an understanding of how the structural remodeling of the heart, a central node in the cardiovascular network, is functionally coupled to the performance of the neuromuscular system (Pelliccia et al., 2025; Squeo et al., 2025). The question of whether the physiological environment that fosters long-term cardiac remodeling is conducive or detrimental to the adaptations required for maximal power development remains unexplored at this systemic, network level.

Therefore, grounded in the principles of NPE (Balagué et al., 2022; Balague et al., 2022; Ivanov, 2021; Ivanov and Bartsch, 2014; Bartsch et al., 2015; Bashan et al., 2012; Garcia-Retortillo and Ch Ivanov, 2025), this study based on our previous work (Papadakis et al., 2025), aimed to address this fundamental gap by quantifying the inter-system coupling between chronic cardiac adaptation and functional anaerobic power in elite soccer players. We sought to determine if LVMI, a key marker of cardiovascular remodeling (Oxborough et al., 2024), independently explains variance in explosive power, as measured by repetitive vertical jump performance (Bishop et al., 2023). Based on the documented interference phenomenon, we hypothesized that a higher LVMI, reflecting a more pronounced endurance-type cardiac adaptation, would be inversely associated with key metrics of anaerobic power. This would reveal a negative coupling within the cardio-neuromuscular network, representing a physiological paradox where the optimization of one system may occur at the functional expense of the other (Gunther et al., 2022).

## 2 Methodology

### 2.1 Participants

This is a secondary NPE-related analysis of data originally collected for a previously published study (Papadakis et al., 2025). Briefly, a cross-sectional, observational study design with criterion sampling was employed to investigate the relationship between LVMI, derived from echocardiography measurements, and indices of explosive anaerobic performance derived from a 15-repetition RVJT. All players completed the same anthropometric, echocardiographic, and RVJT protocol under standardized conditions. To test the hypothesis of the study, 19 adult Greek male Super League professional soccer players (25.66 ± 4.55 years, 1.81 ± 0.07 m, 75.85 ± 7.27 kg, BMI: 23.50 ± 0.48 kg/m^2^, 11.53 ± 3.39 years of training experience) were examined at the beginning of the preseason preparatory period. The inclusion criteria were the participation in international top-level competitions and their involvement in systematic training. Exclusion criteria were the occurrence of severe injury and/or other health problems that did not allow them to participate in their training and competition obligations for a period of 6 months prior to the measurements. All participants provided signed informed consent. The study was conducted following the guidelines of the Declaration of Helsinki and of the Institution’s Research Committee Ethics Code and approved by the Institutional Review Board of the Aristotle University of Thessaloniki, Greece (#281/07.24.25).

### 2.2 Experimental procedures

#### 2.2.1 Echocardiographic measurements

Prior to any functional testing, all participants underwent a clinical screening that included a medical history review and a resting 12-lead electrocardiogram. Cardiac structure and function were then assessed by an experienced cardiologist-ultrasonographer using two-dimensional (2D) echocardiography with a Vivid S70 system (GE Medical; Horten, Norway) equipped with an M5 S phased-array transducer. The examination focused on left and right heart anatomy and function in both systole and diastole. The LVM was specifically quantified from 2D images using the truncated ellipsoid technique, while the modified biplane Simpson’s method was used to calculate the left ventricular ejection fraction (LVEF). To normalize for body size, LVM was indexed to body surface area (BSA) to derive the LVMI (Oxborough et al., 2024; Olsen et al., 2022; Lang et al., 2015; Ahmadi et al., 2023; Nagueh et al., 2016). All acquired images were stored in EchoPAC (version 204) and subsequently analyzed in a randomized sequence by two independent, experienced cardiologists to mitigate bias. The entire procedure and all analyses were conducted in accordance with the established guidelines of the American Society of Echocardiography and the European Association of Cardiovascular Imaging (Nagueh et al., 2016; Heidenreich et al., 2022; Arbelo et al., 2023).

#### 2.2.2 Biomechanical measurements

After the echocardiographic evaluation, each participant performed 15 consecutive maximal effort countermovement jumps on a prototype 1.00 × 1.00 m^2^ K-Force force plate (KINVENT, Biomechanique Montpellier, France) to assess RVJT performance. The primary outcome variables included h_MAX_ and h_AVE_, as well as the average (RSI_AVE_) and maximum (RSI_MAX_) reactive strength index, and relative power output (P_REL_). The relative power output at the first 5 s of the RVJT (P_REL_5s), and the frequency (1/total time to conclude the 15 RVJT; Freq15) were also obtained as indicators of explosive effort to perform the RVJT. During the RVJT, vertical ground reaction forces (V_GRF_) were recorded at a sampling frequency of 1 kHz. The raw force data underwent processing with a low-pass Butterworth recursive filter, with the cut-off frequency set at 20 Hz according to the sum of residual method (Winter, 2009). From the V_GRF_ time series, ground contact time (t_C_) and flight (t_FL_) time were determined for each jump in the RVJT series. Jump height (h_JUMP_) was calculated from t_FL_ (Theodorou et al., 2013), and the reactive strength index (RSI) was determined as the ratio of h_JUMP_ to t_C_ (Panoutsakopoulos et al., 2022). The h_AVE_ and RSI_AVE_ across all 15 jumps were used for the final analysis. The average relative P_REL_ was calculated using the Bosco et al. formula (Bosco et al., 1983). Participants received verbal encouragement to perform each jump with maximal effort throughout the RVJT series.

### 2.3 Statistical analysis: a network physiology of exercise perspective

All analyses were conducted in R (v4.3.1) using *tidyverse, qgraph, bootnet, NetworkComparisonTest*, and *BayesFactor.* Two-sided tests used α = 0.05. For network models, edges were selected via *EBICglasso* with *γ* = 0.50, and inference was supported by non-parametric bootstrapping (Bartsch et al., 2015; The R Development Core Team, 2021; Balague et al., 2020).

#### 2.3.1 Covariate engineering via principal component analysis (PCA)

To control for body size/composition while avoiding multicollinearity, we performed a principal component analysis (PCA) on the correlation matrix of six pre-specified anthropometrics (body mass, height, body-fat percentage, BMI, BSA, lean body mass). The first principal component (PC1), interpreted as a *Size/Composition* factor, was *z*-scored and used as a single covariate in all residualization models, together with *Training Experience* (years). The PCA was run on complete cases for these variables; if data points were missing was present, we applied single-chain predictive mean matching imputation before PCA. Full variance explained and loadings are reported in the Supplement (Jolliffe and Cadima, 2016; Ried et al., 2016; Gonzalez-Gil et al., 2024).

#### 2.3.2 Data preprocessing and node definition

Primary variables (*LVMI, h*_*MAX*_ *, h*_*AVE*_ *, RSI*_*AVE*_ *, RSI*_*MAX*_ *, P*_*REL*_ *, P*_*REL*_ *5s, Freq15*) were residualized on *PC1(Size/Composition)* and *Training Experience* using ordinary least squares. Residuals were *z*-scored and then converted to rank-based normal scores (Blom transformation) to reduce sensitivity to non-normal marginals. These transformed residuals defined the network nodes. The efficacy of this residualization step for each node was audited by inspecting the regression model statistics, including R-squared values (Balague et al., 2022; Balague et al., 2020; Thompson et al., 2017).

#### 2.3.3 Network estimation, stability, and inference

Gaussian Graphical Models were estimated via EBICglasso using *γ* = 0.50 (primary) and *γ* = 0.25 (exploratory). Edge weights are partial correlations conditional on all other variables. Bootstrapped 95% CIs were obtained for edge weights; correlation-stability (CS) coefficients were derived via case-dropping bootstraps. Reliability was assessed with (i) nonparametric bootstrapping (1,000 resamples) to obtain 95% confidence intervals for edge weights and (ii) case-dropping bootstraps (1,000 resamples) to compute the correlation-stability (CS) coefficient for Strength centrality. Centrality was interpreted only when CS exceeded 0.25; if CS was below this threshold, centrality indices were reported descriptively and not used for hypothesis testing (Bartsch et al., 2015; Bashan et al., 2012; Garcia-Retortillo and Ch Ivanov, 2025; Balague et al., 2020; Lehnertz et al., 2020).

#### 2.3.4 Group network comparison

Participants were dichotomized by the clinical LVH threshold (LVMI ≥115 g·m^−2^) to test hypertrophy-linked reconfiguration (Oxborough et al., 2024). The *Network Comparison Test* (NCT) (5,000 permutations) assessed network-structure invariance, global strength (sum of absolute edge weights), and edgewise differences between Normal- and High-LVMI networks, controlling the false discovery rate across edgewise tests. For the NCT, the tuning parameter was set to *γ* = 0.25. This less conservative value was chosen to reduce the risk of estimating empty (zero-edge) networks within the smaller subgroups, which would preclude statistical comparison, while still controlling for spurious connections (Bartsch et al., 2015; Lehnertz et al., 2020; van Borkulo et al., 2023).

#### 2.3.5 Selective coupling index

To directly test the hypothesis that LVMI couples *preferentially* to peak power output over other related metrics, we computed a Selective Coupling Index (SCS) defined as: SCS = abs(w(LVMI-h_MAX_)) − P95[abs(w(LVMI-h_AVE_)), abs(w(LVMI-RSI_AVE_)), abs(w(LVMI-RSI_MAX_)), abs(w(LVMI-P_REL_))], where *w(A–B)* is the EBICglasso partial-correlation edge weight, |·| is absolute value, and P95 is the 95th percentile of the listed values. SCS >0 indicates that LVMI–h_MAX_ exceeds at least 95% of LVMI’s alternative couplings to other jump metrics (Papadakis et al., 2022b; Papadakis et al., 2022c).

#### 2.3.6 Bayesian complement

For the focal LVMI–h_MAX_ pair (after residualization), we computed a Bayesian correlation using a Jeffreys–beta* prior (*r*-scale = 0.333). Bayes factors (*BF*_*10*_ *)* were interpreted alongside network estimates to provide graded evidence at small *n* (Huth et al., 2023; Morey et al., 2015).

#### 2.3.7 Sensitivity analyses

Robustness was evaluated by: (i) re-estimating EBICglasso on the untransformed standardized residuals; (ii) comparing solutions across a *γ* grid (0.25 vs. 0.50); (iii) leave-one-out re-estimation tracking the LVMI–h_MAX_ edge; and (iv) fitting an alternative sparse estimator (*ggmModSelect*) as a specification check (Bartsch et al., 2015; Balague et al., 2020).

#### 2.3.8 Missing data, reproducibility and outliers

Analyses were performed on complete cases. If missingness was detected among analysis variables, we applied single-chain predictive mean matching (m = 1) prior to residualization and archived diagnostics. Following preprocessing, the residualized data for all complete cases were subjected to a multivariate outlier scan using robust Mahalanobis distance (covMcd). All code, random seeds, and session information will be archived with the manuscript on OSF (The R Development Core Team, 2021).

## 3 Results

### 3.1 Participant characteristics

The analysis included a sample of 19 elite male soccer players, who were categorized based on a clinical threshold for left ventricular hypertrophy into a Normal-LVMI (*n* = 8) and a High-LVMI (*n* = 11) group. All primary variables were residualized on a size-composition factor (PC1) and training experience, then standardized and transformed to rank-based normal scores to meet modeling assumptions. A robust Mahalanobis distance scan revealed no significant multivariate outliers in the residualized data (all p > 0.025), indicating high data quality among the complete cases used for network estimation. All subsequent analyses were performed on complete cases. Participant characteristics are detailed in Table 1.

**TABLE 1:** Participant characteristics by left ventricular mass index group.

Variable	Mean ± SD (Overall)	Median [IQR] (Overall)	Mean ± SD (Normal)	Median [IQR] (Normal)	Mean ± SD (High)	Median [IQR] (High)	*p* value	Effect size
Sample size	19	​	8	​	11	​	​	​
BM (kg)	75.85 ± 7.27	74.40 [70.80, 79.60]	74.69 ± 4.46	74.55 [72.35, 77.50]	76.70 ± 8.91	74.40 [70.60, 81.00]	0.589	*g* = −0.26
Height (m)	1.81 ± 0.07	1.82 [1.75, 1.88]	1.79 ± 0.08	1.81 [1.73, 1.84]	1.82 ± 0.07	1.82 [1.76, 1.88]	0.410	*g* = −0.38
BF (%)	11.72 ± 2.18	11.93 [9.96, 13.63]	12.26 ± 1.41	12.60 [10.87, 13.63]	11.33 ± 2.60	10.99 [9.42, 13.53]	0.367	*g* = 0.41
BMI (kg·m^-2^)	23.50 ± 0.48	23.50 [23.30, 23.70]	23.62 ± 0.65	23.80 [23.10, 24.20]	23.41 ± 0.32	23.50 [23.40, 23.60]	0.374	*g* = 0.42
BSA (m^2^)	1.96 ± 0.13	1.96 [1.87, 2.02]	1.94 ± 0.10	1.96 [1.86, 1.99]	1.98 ± 0.14	1.99 [1.87, 2.06]	0.466	*g* = −0.35
LBM (kg)	67.18 ± 6.90	67.02 [63.19, 70.05]	65.65 ± 4.16	65.44 [63.15, 69.14]	68.29 ± 8.39	67.02 [63.30, 70.92]	0.467	*g* = −0.36
h_AVE_ (m)	0.25 ± 0.04	0.24 [0.22, 0.27]	0.27 ± 0.05	0.26 [0.24, 0.29]	0.24 ± 0.03	0.23 [0.22, 0.26]	0.134	*g* = 0.70
**h**_**MAX**_ **(m)**	**0.31 ± 0.05**	**0.31 [0.29, 0.35]**	**0.35 ± 0.04**	**0.35 [0.32, 0.37]**	**0.29 ± 0.04**	**0.30 [0.27, 0.31]**	**0.004**	***g* = 1.51**
P_REL_ (W·kg^-1^)	10.78 ± 0.91	10.70 [10.25, 11.30]	11.10 ± 1.11	11.05 [10.65, 11.62]	10.55 ± 0.71	10.30 [10.20, 11.00]	0.213	*g* = 0.58
RSI_AVE_	1.49 ± 0.20	1.50 [1.39, 1.65]	1.48 ± 0.26	1.51 [1.40, 1.58]	1.50 ± 0.17	1.47 [1.39, 1.65]	0.880	*g* = −0.07
RSI_MAX_	1.86 ± 0.28	1.84 [1.68, 2.07]	1.86 ± 0.29	1.86 [1.74, 2.10]	1.87 ± 0.29	1.84 [1.67, 1.96]	0.976	*g* = −0.01
P_REL_5s (W·kg^-1^)	10.88 ± 0.97	10.80 [10.35, 11.30]	11.30 ± 1.22	11.20 [10.55, 11.73]	10.58 ± 0.64	10.40 [10.25, 11.00]	0.118	*g* = 0.74
Freq15 (Hz)	1.69 ± 0.18	1.72 [1.54, 1.84]	1.61 ± 0.18	1.58 [1.48, 1.73]	1.74 ± 0.18	1.74 [1.63, 1.90]	0.150	*g* = −0.67
TE (years)	11.53 ± 3.39	12.00 [10.00, 14.50]	12.00 ± 3.46	12.50 [9.50, 14.25]	11.18 ± 3.46	10.00 [10.00, 14.50]	0.644	*g* = 0.23

All values are presented as mean and standard deviation (SD), while median values are presented with their respective interquartile ranges (IQR). Effect size is reported as Hedges g. Body mass, BM; Body fat, BF; Body mass index, BMI; Body surface area, BSA; Lean body mass, LBM; Average jump height, h_AVE_; Maximum jump height, h_MAX_; Relative power, P_REL_; Average reactive strength index, RSI_AVE_; Maximum reactive strength index, RSI_MAX_; Relative power 5s, P_REL_5s; Jumping frequency of 15 jumps, Freq15; Training experience, TE.

### 3.2 Covariate engineering via principal component analysis

To control for anthropometrics, a principal component analysis was performed. The first principal component (PC1) explained 68% of the total variance and was retained as a single Size-Composition covariate. This component captured a trade-off between lean size and adiposity, with negative loadings for body mass (−0.47), height (−0.47), BSA (−0.49), and lean body mass (−0.47), and positive loadings for body fat percentage (0.24) and BMI (0.20).

### 3.3 Network estimation and topology

**FIGURE 1:**
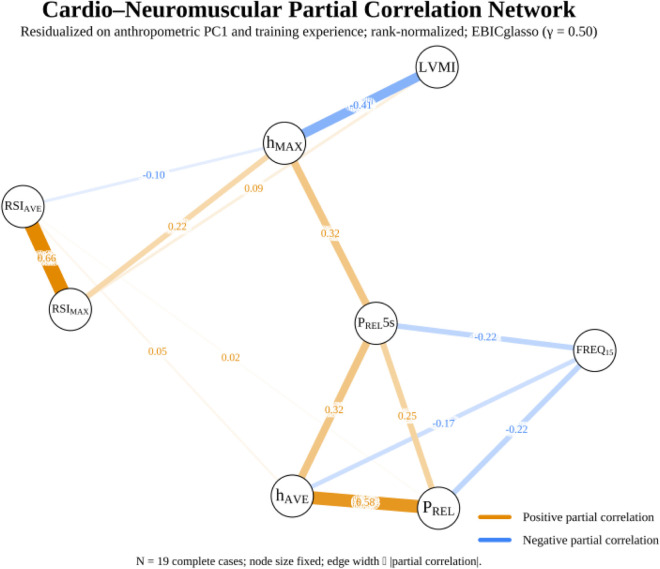
Cardio-neuromuscular partial correlation network (primary).

A Gaussian graphical model was estimated using *EBICglasso* with the primary regularization parameter set to γ = 0.50 (Figure 1). The resulting network was sparse, revealing several physiologically relevant connections. In direct support of our hypothesis, a negative edge was identified between LVMI and h_MAX_ (*p*_cor_ = −0.41). The model also identified strong positive couplings within the neuromuscular subsystem, including between RSI_MAX_ and RSI_AVE_ (*p*_cor_ = 0.66) and between P_REL_ and h_AVE_ (*p*_cor_ = 0.58). Freq15 exhibited modest negative couplings with P_REL_, *p*_cor_ = −0.22 and P_REL_5s, *p*_cor_ = −0.22.

### 3.4 Edge reliability and central stability

Nonparametric bootstrapping (1,000 resamples) confirmed the stability of the estimated edge weights. The case-dropping correlation-stability coefficient for Strength centrality was 0.0, falling below the *a priori* threshold of 0.25. Consequently, centrality indices were deemed unstable in this sample and are not interpreted for hypothesis testing.

### 3.5 Bayesian focal test

A Bayesian correlation test on the rank-normalized residuals of LVMI and h_MAX_ yielded *BF10* = 5.70 (Jeffreys–beta* prior, *rscale* = 0.333), providing moderate evidence for a non-zero negative association. The corresponding Pearson correlation was *r* = −0.57, *p* = 0.012.

### 3.6 Selective coupling test

The Selective Coupling Index (SCS), designed to test if LVMI coupled preferentially to h_MAX_, was SCS = 0.19. While the positive point estimate favors the hypothesis of selective coupling, the 95% bootstrap confidence interval was wide and contained zero (−0.42–0.61), indicating this result was not statistically significant.

### 3.7 Group network comparison by left ventricular hypertrophy status

To test for network differences between the Normal- and High-LVMI groups, the Network Comparison Test (NCT) was performed. An exploratory *γ* of 0.25 was used for this analysis to prevent the estimation of empty graphs in the small subgroups, ensuring a meaningful comparison could be made. The NCT revealed no significant differences between the groups in overall network structure (M-statistic = 0.71, *p* = 0.65), global strength (S-statistic = 1.40, *p* = 0.49), or any individual edge weight.

### 3.8 Sensitivity analyses

**FIGURE 2:**
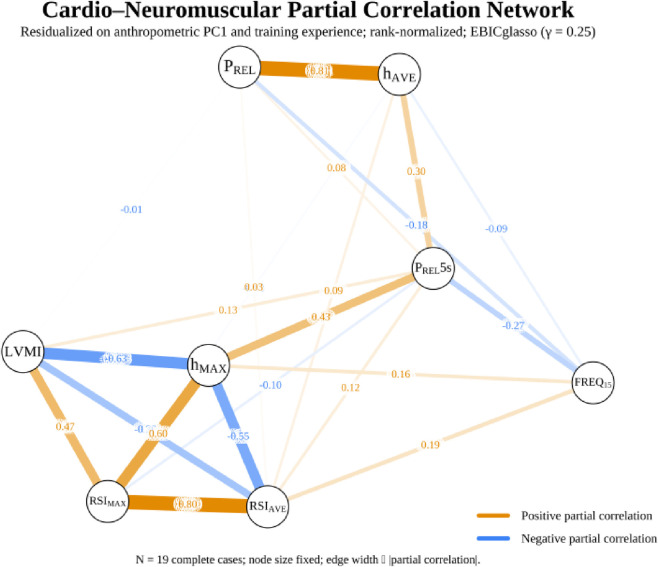
Cardio-neuromuscular partial correlation network (exploratory).

The primary findings remained robust across several sensitivity analyses. Re-estimating the network with *γ* = 0.25 increased edge density but preserved the negative LVMI–h_MAX_ edge and the dominant intra-jump couplings (Figure 2). A leave-one-out re-estimation confirmed that the LVMI–h_MAX_ edge remained consistently negative across all participant subsamples. An alternative sparse estimator (*ggmModSelect*) produced a qualitatively similar network topology, further strengthening confidence in the results.

## 4 Discussion

This study aimed to reframe under the NPE perspective (Balagué et al., 2022; Ivanov, 2021; Balague et al., 2020) the siloed approached of interference of concurrent training between cardiovascular and neuromuscular systems (Pelliccia et al., 2025; Squeo et al., 2025). More specifically, we sought to quantify the inter-system coupling between chronic cardiac adaptation and functional anaerobic power in elite soccer players. Therefore, it was hypothesized that higher LVMI would be inversely associated with repetitive vertical jump performance.

Our model captures static conditional associations based on inter-individual variance. It does not identify direction, feedback, or within-athlete dynamics. Crucially, in non-ergodic systems, between-person associations do not necessarily reflect within-person relations; this inherent limitation restricts the causal claims that can be drawn from a single cross-sectional sample (Saqr et al., 2024; Bialek et al., 2024; Mangalam and Kelty-Stephen, 2022; Molenaar, 2008; Molenaar and Campbell, 2009; Mangalam and Kelty-Stephen, 2021; Hunter et al., 2024). Our primary finding, which was validated across multiple statistical approaches, confirmed our hypothesis. After controlling for size/composition and training experience, results revealed that greater LVMI relates to lower maximal jump height in the multivariate cardio-neuromuscular network context. This primary finding was notably robust; the negative network edge between LVMI and h_MAX_, which represents a unique association conditional on all other variables, persisted even when the network was re-estimated under less stringent, exploratory conditions (*γ* = 0.25) that allowed for a greater number of connections. This association was even corroborated from the Bayesian check of moderate, non-zero association and the coherent neuromuscular cluster of strong positive coupling of RSI and power related metrics (i.e., RSI_MAX_↔RSI_AVE_; P_REL_↔h_AVE_). The developed neuromuscular cluster behaved as expected in terms of the SSC constructs: the h_AVE_ correlated strongly with P_REL_ and P_REL_5s, and RSI_AVE_ correlated strongly with RSI_MAX_, findings that are consistent with previous literature in elite soccer (Akyildiz et al., 2022; Bishop et al., 2022; Munoz-Gracia et al., 2025). The LVMI-h_MAX_ edge stood out as selectively negative, whereas LVMI showed no comparable negative coupling to h_AVE_ or RSI metrics, indicating a relationship focused on the velocity-dependent, peak end of the force–velocity spectrum rather than on fatigue-resistant averages (Oxborough et al., 2024; Egan and Sharples, 2023; Solberg et al., 2025). Parallel to the previous, Freq15 displayed negative partial edges to P_REL_ and P_REL_5s, a pattern compatible with the known cadence–impulse trade-off in repeated-jump tasks, where higher cadence accompanies lower per-jump impulse and power (Deely et al., 2022; Akyildiz et al., 2022; Stratford et al., 2020; Theodorou et al., 2013; Bosco et al., 1983). Results also revealed no evidence that overall network structure or global strength differs between “normal” vs. “high” LVMI groups in this sample.

### 4.1 Context with prior literature

Due to the novelty of the approach, results will be discussed in terms of NPE and in direct comparison of a previous work that examined the cardio-neuromuscular performance paradox by the traditional linear modeling (Papadakis et al., 2025). We believe that this discovery moves beyond the simple linear correlation and provides a more comprehensive perspective by revealing a systemic trade-off within the human physiolome. This trade-off should not be perceived as a flaw, but instead as an intricate balancing act between endurance and power, as an emergent property of a highly integrated and optimized physiological network (Balagué et al., 2022; Balague et al., 2022; Ivanov, 2021; Ivanov and Bartsch, 2014; Ivanov et al., 2021a).

In this study we challenged the previous siloed concept of athletic adaptations (Papadakis et al., 2025) by highlighting the interconnected and sometimes antagonistic nature of physiological systems (Balagué et al., 2022; Balague et al., 2020; Lehnertz et al., 2020; Ivanov et al., 2021a). The observed inverse relationship between LVMI and explosive power parallels what the current interference theory depicts in concurrent training model (Schumann et al., 2022; Vechin et al., 2021; Huiberts et al., 2024; Arslan et al., 2025; Malamud and Smukas, 2025; Gomes et al., 2025; Wang and Bo, 2024; Chen et al., 2024; Blechschmied et al., 2024; Thomakos et al., 2023; Seipp et al., 2022; Petre et al., 2018; Edwards et al., 2023), but it reframes it at the systemic level.

Traditionally, this effect is explained at a molecular level, where the signaling pathways governing endurance adaptations (e.g., AMP-activated protein kinase [AMPK]) are thought to inhibit those responsible for hypertrophy and power (e.g., mammalian target of rapamycin [mTOR]) (Egan and Sharples, 2023; Furrer and Handschin, 2024; Levitt et al., 2022; Steinberg and Hardie, 2023). By challenging soccer’s unique chronic hybrid physiological demands that induce profound eccentric and concentric cardiac remodeling (Pelliccia et al., 2023; Oxborough et al., 2024), not related to pathology (Baba Ali et al., 2024; Albaeni et al., 2021), we interpret the interference effect in a broader way that goes beyond the molecular signaling pathways (Schumann et al., 2022; Petre et al., 2021) to a whole organism negotiation.

This negotiation between chronic aerobic and anaerobic demands of elite soccer (Hostrup and Bangsbo, 2023), induced increased myocardial mass, which even when it was scaled for body surface area (Oxborough et al., 2024), contributed to the load that the neuromuscular system had to overcome during repetitive jumps. Evidence of that is that in our cohort, athletes with greater LVMI exhibited lower peak vertical jump height, despite similar body masses, suggesting that the trade-off extends beyond mere biomechanics (Boraczynski et al., 2020; Hostrup and Bangsbo, 2023). The reduced jump frequency and power output we observed in athletes with higher LVMI suggest that while the cardiovascular system adapts to enhance aerobic efficiency, it may inadvertently tax the very systems responsible for the rapid, game-deciding bursts of power (Gunther et al., 2022).

At the neuromuscular subnetwork level, the strong positive partial associations between average jump height and relative power outputs (h_AVE_ with P_REL_ and P_REL_5s) align with SSC efficiency and power constructs used in standard CMJ and DJ profiling frameworks (Hostrup and Bangsbo, 2023; Bishop et al., 2022; Theodorou et al., 2013; Bishop et al., 2023; Munoz-Gracia et al., 2025). This clustering reinforces construct validity for the test battery in elite soccer settings (Bishop et al., 2022; Munoz-Gracia et al., 2025). Similarly, both RSI_AVE_ and RSI_MAX_ also showed a tight positive link, aligning with evidence that RSI-type indices co-vary and respond to changes in plyometric loading and neuromuscular status (Akyildiz et al., 2022; Bishop et al., 2022; Munoz-Gracia et al., 2025). On the other hand, F_REQ_15 displayed negative partial edges with power-related nodes such as P_REL_ and P_REL_5s. This pattern is compatible with the mechanical trade-off seen in repeated-jump tasks, where higher cadence is typically achieved with lower per-jump impulse and power, as described in method work on repeated-jump testing and continuous jumping mechanics (Boraczynski et al., 2020; Deely et al., 2022; Akyildiz et al., 2022; Theodorou et al., 2013; Bosco et al., 1983).

It is evident that the documented relationship between LVMI and RVJT was specific to h_MAX_ and not to other jump power metrics such as h_AVE_ or RSI, that provide a focused view of network’s interaction. It is reported that h_MAX_ reflects peak explosive output and the capacity to generate force rapidly in a single effort (Bishop et al., 2022; Theodorou et al., 2013), while h_AVE_ and RSI reflect elements of fatigue resistance and SSC efficiency across repeated efforts (Akyildiz et al., 2022; Munoz-Gracia et al., 2025). Taken all together and the present deficit in h_MAX_ suggests that high-LVMI network state does not globally depress neuromuscular function. It actually points to the fact of a selective constrain on the velocity component of the force-velocity spectrum that affects the peak explosive capacity while preserves the repeated submaximal outputs (Egan and Sharples, 2023; Solberg et al., 2025). Such specificity in adaptations is in agreement with complex systems behavior in which a uniform suppression is dropped in favor of a targeted and dynamic network adjustment (Haken, 2006).

Conversely, while our cross-sectional design precludes causal inference (Savitz and Wellenius, 2023), the concept of coupling implies that influences are not unidirectional. While our network analysis suggests that an enlarged ventricle is inversely coupled with jump height, this should not be interpreted as simple mechanical causation. It is equally plausible that a neuromuscular system chronically biased toward explosive, high-force contractions might generate transient pressure overloads that contribute to concentric cardiac remodeling over time (Green et al., 2024; Churchill et al., 2021; Savitz and Wellenius, 2023). This perspective aligns with evidence of dynamic cardiorespiratory coordination during and after exercise, where the heart, lungs, and muscles operate as a tightly integrated unit rather than as independent modules (Balague et al., 2016; Garcia-Retortillo et al., 2017; Schulz et al., 2013).

Such an understanding corroborates the findings of previous work where it was demonstrated an isolated robust cardiac remodeling and powerful neuromuscular performance (Papadakis et al., 2025). The current NPE approach, where we treated LVMI and neuromuscular outputs as dynamic interacting nodes, assessed whether the involved systems may influence each other under a more comprehensive systems-level understanding. From this vantage point, we showed that LVMI and RVJT performance are not independent, but are coupled in a dynamic cardio muscular network where their inverse quantified relationship was not fully captured when these systems were examined in isolation (Papadakis et al., 2025; Balagué et al., 2022; Balague et al., 2022; Ivanov and Bartsch, 2014; Bartsch et al., 2015; Bashan et al., 2012; Balague et al., 2020; Ivanov et al., 2021a; Balague et al., 2015; Ivanov et al., 2017; Ivanov et al., 2021b).

### 4.2 Mechanistic hypotheses of the cardio-neuromuscular paradox

The NPE approach revealed a specific LVMI-power relationship where the observed tradeoff was isolated to h_MAX_ pointing towards to interconnected involved mechanisms that extend beyond simple biomechanical load. It seems that the geometry of the LVH is a factor that is affected by the hybrid load of elite soccer which promotes a mix of eccentric (volume-induced) and concentric (pressure-induces) cardiac remodeling (Johnson et al., 2023; Baba Ali et al., 2024; Mihl et al., 2008; Maxwell and Oxborough, 2025; Rossi et al., 2024). As such, the systemic shift towards endurance-optimized training—specifically the development of an eccentric phenotype—is a crucial adaptation for effective cardiac output. However, this shift may create a physiological milieu less conducive to the rapid, high-frequency motor unit recruitment required for maximal explosive power, as assessed by the RVJT (Lewis et al., 2012; Di et al., 2024; Di et al., 2025; Flanagan et al., 2023; Kim et al., 2024). Therefore, the noted tradeoff may have been originated not just from the added mass, but from the underlying systemic adaptations that the specific phenotype remodeling represents.

It is possible that the proposed root of the suggested cardo-neuromuscular paradox to be traced to the autonomic nervous system. As suggested earlier, this specific phenotype may develop a high LVMI which is functionally coupled with enhanced parasympathetic modulation and vagal dominance, a common characteristic of high-level endurance athletes (Kim et al., 2024; Lipka et al., 2025; Tekin et al., 2025; Gigante et al., 2025; Fuentes-Barria et al., 2025). This autonomic status though, while it is beneficial for cardiac efficiency and recovery, its primary action is fundamentally antagonistic to the massive sympathetic outflow required to orchestrate maximal-intensity, explosive efforts like the ones observed in repetitive jumps (Ishida et al., 2021; Le et al., 2020). Based on this explanation, the observed decline in h_MAX_ and under the NPE lens could therefore not just reflect a muscular limitation, but a systemic tradeoff at the level of the autonomic nervous system.

Following the same line of thought, the noted interference may extend to the muscular level itself where the cardiac remodeling favors a shift towards to a more oxidative muscle fiber phenotype (Huiberts et al., 2024; Lundberg et al., 2022). Such an adaptation will increase the fatigue resistance, but it may come to a cost of the performed jumping activities that relate to force-generating capacity and contraction velocity of type IIx fibers (Methenitis et al., 2025; Sterczala et al., 2024). Subsequently, any high-LVMI state could be an indication of a reorganization of the system towards to a prioritized fatty acid oxidation and metabolic efficiency over the rapid glycolytic flux necessary for explosive, anaerobic power (Hearris et al., 2018; Mora-Fernandez et al., 2025).

### 4.3 Methodological reflections: a conceptual bridge to true network physiology

A primary contribution of this investigation is the application of a Gaussian graphical model, which advances our understanding of the “cardio-neuromuscular paradox” beyond the linear relationships reported in our prior work. In that antecedent study, we established a robust, group-level inverse association between LVMI and h_MAX_ using conventional hierarchical multiple regression (Papadakis et al., 2025). The current network analysis builds significantly on this foundation. It demonstrates that the negative LVMI - h_MAX_ partial correlation (*p*_*cor*_ = −0.41) is not a statistical artifact of other neuromuscular couplings, such as the strong, positive intra-system link between h_AVE_ and P_REL_ (*p*_*cor*_ = 0.58) (Papadakis et al., 2025).

This triangulation of evidence (Gutierrez et al., 2025; Hammerton and Munafo, 2021), however, also illuminates a shared and fundamental limitation: both studies are cross-sectional and infer physiological structure from group-pooled, inter-individual variance (Savitz and Wellenius, 2023). This methodological approach is in direct tension with the core tenets of Network Physiology, which emphasizes the analysis of intra-individual dynamics captured via dense time-series data (Balague et al., 2022).

### 4.4 Non-ergodicity and the limits of static networks

In a non-ergodic biological system, the variance observed between individuals at a single point in time (inter-individual) does not necessarily map onto the dynamic processes occurring within a single individual over time (intra-individual) (Saqr et al., 2024; Bialek et al., 2024; Mangalam and Kelty-Stephen, 2022; Molenaar, 2008; Molenaar and Campbell, 2009; Mangalam and Kelty-Stephen, 2021; Hunter et al., 2024). Therefore, the negative LVMI - h_MAX_ edge identified in our group-level network, while statistically robust across two different methodologies (Papadakis et al., 2025), must be interpreted with caution. It represents a population-level trend, but it may be a “statistical artifact” of pooling heterogeneous individuals rather than a true representation of an underlying physiological structure present within any single athlete (Saqr et al., 2024; Bialek et al., 2024; Mangalam and Kelty-Stephen, 2022; Molenaar, 2008; Molenaar and Campbell, 2009; Mangalam and Kelty-Stephen, 2021; Hunter et al., 2024). To be explicit, our findings cannot infer causality (Savitz and Wellenius, 2023) or imply that as a single athlete’s LVMI increases during a training macrocycle, their h_MAX_ will necessarily decrease. That is a dynamic, intra-individual hypothesis that our static, cross-sectional data are fundamentally incapable of testing.

### 4.5 Reframing this study’s contribution

Given this context, the primary contribution of this manuscript is not to provide a definitive map of cardio-neuromuscular structure. Instead, this study serves as a conceptual and methodological bridge. By applying NPE principles to a traditional, non-time-series dataset, we achieve two goals: a) we use a multivariate network model to refine a novel finding, the specificity of the LVMI - h_MAX_ trade-off, which was previously only identified with simpler, linear models (Papadakis et al., 2025); b) we use our own “robust” group-level data as a case study to critically reassess the limitations of conventional approaches and to advocate for the correct level of analysis that NPE demands (Balague et al., 2022).

### 4.6 Practical implications

A practical implication of this study is the support for individualized monitoring of adaptation trade-offs in elite soccer, with routine field measures linked to decision making in applied environments (Haken, 2006; Gill et al., 2023; Stellingwerff et al., 2025). The use of RVJT as an athlete’s metric does more than checking a single quality, as it provides different optics into how the involved systems are balanced at that time (Suarez-Arrones et al., 2020; Bishop et al., 2022; Haken, 2006; Gill et al., 2023; Stellingwerff et al., 2025). For example, a decline in peak output that is not explained by injury or acute fatigue can, under an NPE perspective, signal a systemic reorganization that prioritizes endurance-oriented capacity over peak explosiveness (Suarez-Arrones et al., 2020; Bishop et al., 2022; Balagué et al., 2022; Balague et al., 2022; Lehnertz et al., 2020; Ivanov et al., 2021a; Haken, 2006; Ivanov et al., 2021b; Gill et al., 2023; Stellingwerff et al., 2025; Pol et al., 2020). In such cases, coaches can modulate training by alternating mesocycles with distinct cardiovascular and power emphases, shifting selected microcycles from volume to velocity, or inserting brief high-load neuromuscular sessions during aerobic phases rather than layering everything concurrently (Hostrup and Bangsbo, 2023; Wang and Bo, 2024; Blechschmied et al., 2024; Feuerbacher et al., 2025). This level of precision aligns with the demands of contemporary sports, where marginal gains influence outcomes and define success (Hostrup and Bangsbo, 2023; Stellingwerff et al., 2025; Nassis et al., 2020).

### 4.7 Strengths and limitations

One key strength that needs to be highlighted is the multilevel statistical approach. We combined regression modeling, principal component analysis, covariance adjustment for anthropometric variables and years of training experience in an attempt to reduce confounder and enhance interpretability and replicability. Additionally, our residualization audit confirmed that these covariates were meaningfully controlled for, thereby isolating the inter-system coupling more effectively. This triangulation aimed to support the presence of coupling between cardiac structural adaptation and functional neuromuscular capacity (Gutierrez et al., 2025; Hammerton and Munafo, 2021; Murphy et al., 2025; Hecksteden et al., 2022a). The elite status of the cohort (e.g., Super League professionals with >11 years of training) adds translational and ecological value. At such high level of performance, these players operate near their limits of human performance where even small physiological shifts carry substantial implications for their competitive performance, that simple reductionistic ways could not capture (Balagué et al., 2022; Balague et al., 2020).

This study is not free of limitations. Sample size is modest, something common in elite sports research that constrains precision and replicability (Murphy et al., 2025; Hecksteden et al., 2022b). While the sample size limited the stability of global network metrics, the robustness of our primary finding was confirmed through multiple sensitivity analyses and a diagnostic scan that revealed no influential multivariate outliers. In addition, this study is not a Network Physiology investigation in the strict methodological sense. We did not collect concurrent time-series signals from multiple organ systems. We did not apply multiplex network analytics or dynamic coupling metrics such as transfer entropy. As a result, coupling is inferred from cross-sectional proxies rather than quantified directly (Balagué et al., 2022; Balagué et al., 2024; Ivanov and Bartsch, 2014; Ivanov et al., 2016; Bartsch et al., 2015; Balague et al., 2020; Ivanov et al., 2021a; Ivanov et al., 2021b; Balagué et al., 2013; Ivanov et al., 2001).

We acknowledge the cross-sectional design and the sample size as notable constraints under an NPE lens, but the value of this work lies in serving as a conceptual bridge. By applying NPE principles to reinterpret a classic sports science problem, we highlight a previously unquantified cardio-neuromuscular trade-off that aligns with NPE theory. Prior work has shown that an NPE perspective can generate testable hypotheses and can reframe isolated adaptations as products of interdependent network processes. That contribution justifies publication as a concept paper that advances the field’s boundaries (Balagué et al., 2022; Balague et al., 2022; Ivanov, 2021; Garcia-Retortillo and Ch Ivanov, 2025; Balague et al., 2020; Lehnertz et al., 2020; Ivanov et al., 2021a; Jiang et al., 2021; Rizzo et al., 2020; Lin et al., 2020; Barajas-Martinez et al., 2020; Balagué et al., 2020b). This study took a familiar problem and reframed it through the lens of system-level interaction. Reframing from observation to interpretation is essential for building the physio-logical architecture that NPE demands. It is a call for future researchers to move beyond isolated biomarkers and to examine how training, adaptation, and performance emerge from the collective behavior of integrated systems (Papadakis et al., 2022b; Papadakis et al., 2022c; Garcia-Retortillo and Ch Ivanov, 2025; Jiang et al., 2021; Rizzo et al., 2020; Lin et al., 2020; Barajas-Martinez et al., 2020; Stamatis et al., 2023; Stamatis et al., 2024; Held et al., 2023; Thomas, 2022; Romero-Ortuno et al., 2021).

### 4.8 Future directions: a call for intra-individual time-series

This critical reframing leads to a more specific and urgent call for future research. To truly test the “cardio-neuromuscular paradox,” the field must move from inter-individual snapshots to intra-individual, longitudinal (*n*-of-1) designs (Saqr et al., 2024; Bialek et al., 2024; Mangalam and Kelty-Stephen, 2022; Molenaar, 2008; Molenaar and Campbell, 2009; Mangalam and Kelty-Stephen, 2021; Hunter et al., 2024). Such studies must track key variables concurrently and over time within the same athletes. This would involve, for example, measuring cardiac remodeling via validated echocardiographic techniques (Baba Ali et al., 2024; Oxborough et al., 2024) at the start, middle, and end of a season, alongside weekly or bi-weekly force plate assessments of neuromuscular performance (Bishop et al., 2022; Bishop et al., 2023).

Only with such dense, multivariate time-series data can we apply the proper analytical tools of network science, such as transfer entropy or time-varying network models, to quantify the dynamic, directional interplay between systems (Papadakis et al., 2022b; Papadakis et al., 2022c; Garcia-Retortillo and Ch Ivanov, 2025). This is the best suited methodology that can determine if the LVMI - h_MAX_ trade-off is a genuine, dynamic physiological process within an athlete or merely a statistical curiosity of group-level analysis.

It is of paramount importance to the solidification of the NPE perspective that future studies extend this foundation with designs that can test causality. Prospective work should track athletes over time, recruit female and male cohorts, span competitive tiers, and secure sample sizes that improve precision and generalizability (Gutierrez et al., 2025; Hammerton and Munafo, 2021; Hecksteden et al., 2022a; Hecksteden et al., 2022b). Ultimately, the path forward is to integrate high-resolution, multi-system physiological data with the sophisticated analytical tools of network science to truly map the dynamic interplay that governs the limits of human performance; candidate signals may include ECG-derived heart rate variability, surface EMG, respiration, kinetics, and kinematics. Methods such as multiplex and time-varying networks, transfer entropy, and information-theoretic metrics can quantify directionality and state-dependent coupling *in vivo*, a goal now feasible with advances in wearables and computational modeling (Balagué et al., 2022; Balague et al., 2022; Balagué et al., 2024; Ivanov, 2021; Ivanov and Bartsch, 2014; Bartsch et al., 2015; Balague et al., 2020; Ivanov et al., 2021a; Balagué et al., 2013).

## 5 Conclusion

In summary, this study presents initial empirical evidence of a cardio–neuromuscular network, characterized by a negative coupling between left ventricular structural adaptation and explosive power, persisting even after adjustment for body size and training experience. When interpreted through the lens of Network Physiology, athletic adaptation emerges as a set of interdependent processes rather than distinct, parallel outcomes shaped by training history and physiological constraints. This perspective challenges siloed assessments and supports integrative profiling that acknowledges trade-offs inherent to elite performance.

## Data Availability

Publicly available datasets were analyzed in this study. This data can be found here: https://www.doi.org/10.17605/OSF.IO/VJGT3.
